# Extending the minimal model of metabolic oscillations in *Bacillus subtilis* biofilms

**DOI:** 10.1038/s41598-020-62526-6

**Published:** 2020-03-27

**Authors:** Ravindra Garde, Bashar Ibrahim, Stefan Schuster

**Affiliations:** 1 Department of Bioinformatics, Matthias Schleiden Institute, Friedrich Schiller University Jena, Ernst-Abbe-Platz 2, 07743 Jena, Germany; 2 Max Planck Institute for Chemical Ecology Hans-Knöll Str. 8, 07745 Jena, Germany; 3 Centre for Applied Mathematics and Bioinformatics, and Department of Mathematics and Natural Sciences Gulf University for Science and Technology, Hawally, 32093 Kuwait

**Keywords:** Biochemical networks, Differential equations

## Abstract

Biofilms are composed of microorganisms attached to a solid surface or floating on top of a liquid surface. They pose challenges in the field of medicine but can also have useful applications in industry. Regulation of biofilm growth is complex and still largely elusive. Oscillations are thought to be advantageous for biofilms to cope with nutrient starvation and chemical attacks. Recently, a minimal mathematical model has been employed to describe the oscillations in *Bacillus subtilis* biofilms. In this paper, we investigate four different modifications to that minimal model in order to better understand the oscillations in biofilms. Our first modification is towards making a gradient of metabolites from the center of the biofilm to the periphery. We find that it does not improve the model and is therefore, unnecessary. We then use realistic Michaelis-Menten kinetics to replace the highly simple mass-action kinetics for one of the reactions. Further, we use reversible reactions to mimic the diffusion in biofilms. As the final modification, we check the combined effect of using Michaelis-Menten kinetics and reversible reactions on the model behavior. We find that these two modifications alone or in combination improve the description of the biological scenario.

## Introduction

Biofilms, a complex aggregation of cells embedded in a polysaccharide matrix, have been of interest for a long time in history – right when Antoine van Leuwenhoek examined a scraping of his tooth plaqueunder a microscope that he had built^1^. Since then, our understanding of biofilms has greatly broadened. We now know that living as a close aggregation provides several advantages to bacteria, such as the efficient distribution of macronutrients, removal of waste products, defense from chemical stress, and better gaseous exchange in the case of pellicle biofilms on the surface of liquids^2,3^. Biofilms are an elaborate system of coexisting individual cells that exhibit several social dynamics, including the division of labour^4–7^.

In experiments using a microfluidics chamber, oscillations were observed in the growth of *Bacillus subtilis*^4^ which was supplied with glutamate on one end of the chamber while the waste products of the biofilm were washed off at the other end at a constant rate. The peripheral cells of the biofilm have the advantage of direct access to glutamate. On the downside, they lose small molecules like ammonia which is an important source of nitrogen^8^. Cells in the interior, on the other hand, depend on the leftover glutamate that diffuses inwards in the biofilm, but do not lose gaseous molecules as rapidly as the peripheral cells. Thus cells in different regions of the biofilm will likely exhibit differences in their metabolic behavior^7^. The interior cells produce ammonia, which is required to produce glutamine. Glutamine is a proxy for the protein content and thus for the growth of the biofilm^9^. The peripheral cells depend on the ammonia diffusing from the interior for their growth. Thus, interior cells control the growth of the peripheral cells by monopolizing ammonia supply.

When the peripheral cells grow fast, the interior cells start to starve of glutamate, leading to a decline in the production of ammonia. As a result, the growth rate of the peripheral cells drops until the interior obtains enough glutamate. This oscillating system has not only been studied experimentally but also modeled by Liu *et. al*.^4^, using six ordinary differential equations (ODEs) – two for glutamate and the ribosomes (a proxy for cellular machinery) in the two regions of the biofilm each, the enzyme glutamate dehydrogenase and ammonia.

Another approach to modeling this system is using partial differential equations (PDEs), as shown by Bocci *et. al*. in their model that uses four variables instead of six^10^. PDEs are used to simulate the gradient of ammonia and glutamate, and two more ODEs describe the dynamics of housekeeping proteins and the enzyme glutamate dehydrogenase (GDH). In our approach, we focus solely on the two metabolites: glutamate and ammonia, and thus describe the same process in a minimal model^11^. We did this by employing a previously known minimal oscillator involving three ODEs, proposed by Wilhelm and Heinrich, aimed to describe chemical reactions^12^. The three ODEs in variables *X*, *Y*, and *Z* in the minimal oscillator corresponded, in our adapted model, to glutamate in the periphery (*G*_*p*_), interior (*G*_*i*_) and ammonia (*A*) respectively and were found to adequately describe the biofilm oscillations^11^. The system of equations of the basic model (BM) is as follows:

BM1 $$\frac{d{G}_{p}}{dt}={k}_{1}{G}_{E}{G}_{p}-\,{k}_{4}{G}_{p}-{k}_{2}A{G}_{p}$$

BM2 $$\frac{dA}{dt}=-\,{k}_{3}A\,+\,{k}_{5}{G}_{i}$$

BM3 $$\frac{d{G}_{i}}{dt}={k}_{4}{G}_{p}\,-\,{k}_{5}{G}_{i}$$

The parameter names and values are listed in Table 1. Glutamate is the focal amino acid and supplied in the experimental setup. Hence, its external concentration is represented by a constant term (*G*_*E*_). Its concentration within the cells in the periphery of the biofilm is dynamic and is represented by the variable *G*_*p*_. It is plausible to assume a positive feedback of *G*_*p*_ on itself (*k*_1_*G*_*E*_*G*_*p*_) because as more glutamate is taken up, more proteins are produced, including membrane proteins, which help take up more glutamate. It then diffuses to the interior cells (with rate *k*_4_*G*_*p*_) and, there, it is represented by *G*_*i*_. We distinguish the same metabolite using two different variables based on their location in the biofilm since these regions use glutamate for different purposes. Interior cells produce ammonia using glutamate (rate *k*_5_*G*_*i*_) since that produced by the periphery is lost to the environment and is a waste of nitrogen. Peripheral cells use the ammonia diffusing out from the interior in addition to the glutamate supplied in the microfluidics chamber to produce biomass (*k*_2_*AG*_*p*_)^11^. However, the loss of ammonia due to diffusion to the environment (*k*_3_*A*) is much greater than its consumption to produce biomass; hence the term *k*_2_*AG*_*p*_ is neglected in equation BM2.

**Table 1:** List of all parameters for all the models.

Parameter	Symbol	Value with unit
Glutamate concentration in the environment	*G*_*E*_	30 mmol/l
Rate constant of glutamate diffusion from the environment to biofilm	*k*_1_	0.34 (mmol/l* h)^−1^
Biomass formation coefficient	*k*_2_	5.3 (mmol/l* h)^−1^
Rate constant of ammonia diffusion^20^	*k*_3_	4 h^−1^
Rate constant of glutamate diffusion within biofilm^21,22^	*k*_4_	2 h^−1^
Ammonia production coefficient	*k*_5_	2.3 h^−1^
Michaelis-Menten constant	*K*_*m*_	1100 mmol/l
Maximum glutamate uptake rate at saturating concentration	*V*_*max*_	1400 mmol/lh

This model describes all these processes as irreversible, which is quite unrealistic from a biological point of view. Wilhelm and coworkers^13^ did present a reversible version of their oscillator. However, they considered all reactions to be reversible and assumed that the reverse reactions are considerably (10×) slower than the forward reactions, which may not be applicable for all biological scenarios. In particular, the diffusion constant is usually the same for either direction of diffusion.

In this study, we develop four sequels of the basic model to describe the oscillations in the system much more realistically, without compromising too much on minimalism. We first introduce a gradient of metabolites from the center of the biofilm to the periphery using six ODEs (Model 6ODE). Subsequently, we use realistic Michaelis-Menten kinetics instead of mass-action kinetics in addition to including some reversible reactions (Model RMM). Finally, we check the effect of using Michaelis-Menten kinetics (Model MMK) or reversible reactions (Model R) individually in two distinct versions on the model behavior.

## Results and Discussion

### Towards a continuous diffusion gradient within the biofilm

Having discrete interior and peripheral regions in a biofilm is quite adequate in the minimalist philosophy, but in a realistic scenario, there is no rigid boundary separating the interior from the periphery. Instead, there is a gradient of substances, the concentrations of which vary with the distance from the biofilm center. This is an important feature to test since it has not been considered by the BM^11^. Bocci *et. al*. have already modeled these gradients using PDEs^10^.

**Figure 1:**
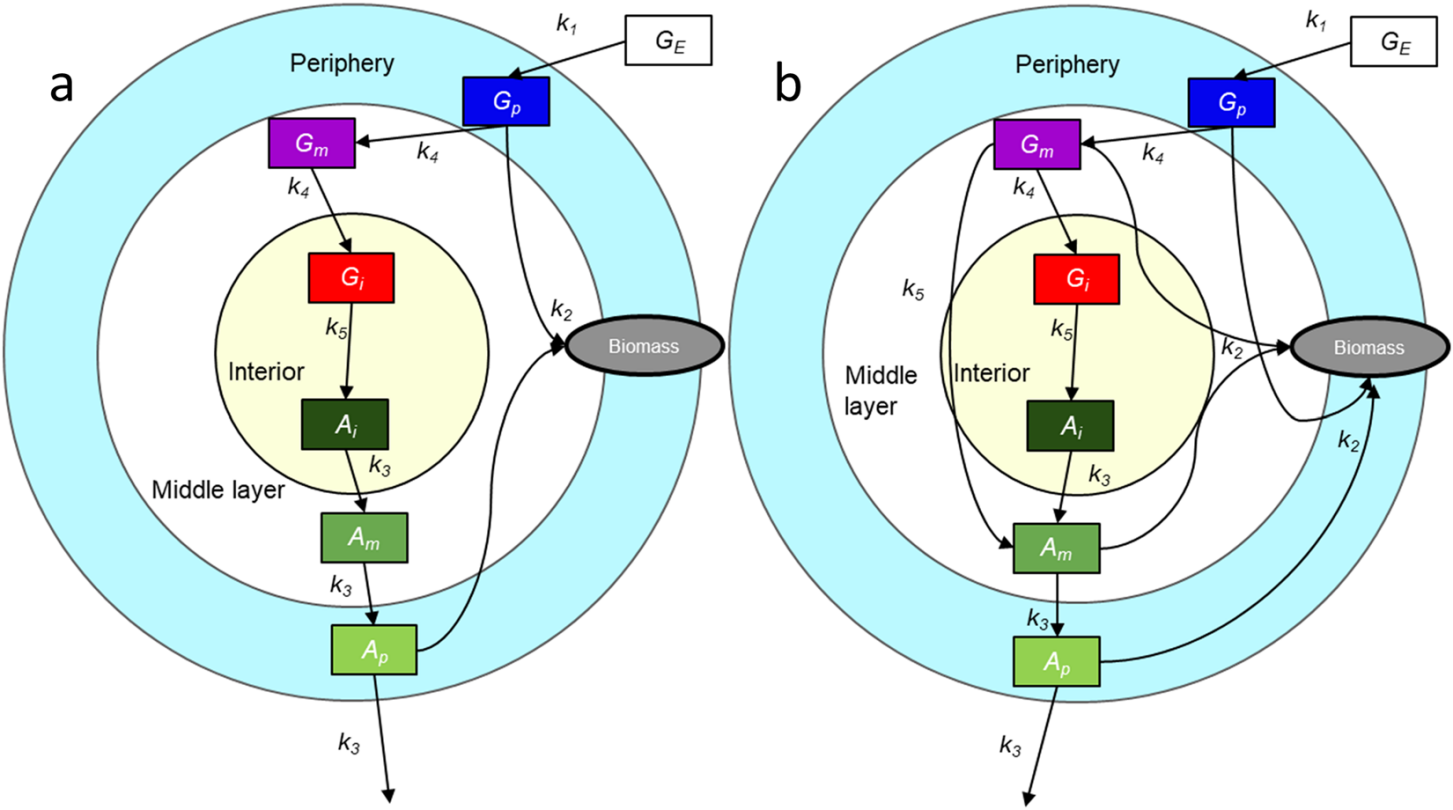
Overview of the six-variable models. (**a**) Simple 6 ODE model (s6ODE): Additional transitional layer between periphery and interior (*G*_*m*_) connected to *G*_*p*_ and *G*_*i*_ through simple diffusion (*k*_4_*G*_*p*_ and *k*_*4*_*G*_*m*,_ respectively). Ammonia is represented by three different variables *A*_*i*_, *A*_*m*,_ and *A*_*p*_ corresponding to each of the three layers also connected through simple diffusion (*k*_3_*A*_*i*_ and *k*_3_*A*_*m*_). **(b)** Complex 6 ODE model (c6ODE): *G*_*m*_ serves not only as a physical but also a functional transitional layer, since it contributes to ammonia production (*k*_5_*G*_*m*_) and to biomass production (*k*_2_*A*_*m*_*G*_*m*_) in addition to being connected through diffusion as in s6ODE. For values of parameters, see Table 1.

In our model, we restrict ourselves to ODEs since PDEs are computationally expensive. This can be done by adding several layers (based on ODEs) to the biofilm. As a first step in this regard, we consider one more layer, represented by *G*_*m*_, at the interface between *G*_*i*_ and *G*_*p*_ (see Fig. 1a,b). Furthermore, the BM only uses one variable (eq. BM2) to describe the dynamics of ammonia with the assumption that diffusion does not play a role in its dynamics because ammonia diffuses fast. We aim to test this assumption by using three separate ODEs for ammonia in the three layers of the model biofilm, namely, *A*_*i*_, *A*_*m*,_ and *A*_*p*_. We further subdivide this model into two categories based on the function of the middle layer. Since the layer is added between the interior and periphery, it can either act simply as a transitional layer (referred to as s6ODE model, Fig. 1a) or it could have a more complex function (referred to as c6ODE model, Fig. 1b) as a layer that contributes to biomass formation (like the periphery) and also ammonia production (like the interior).

1a $$\frac{d{G}_{p}}{dt}={k}_{1}{G}_{E}{G}_{p}-\,{k}_{4}{G}_{p}-{k}_{2}{A}_{p}{G}_{p}$$

1b $$\frac{d{A}_{i}}{dt}=-\,{k}_{3}{A}_{i}+\,{k}_{5}{G}_{i}$$

1c $$\frac{d{A}_{m}}{dt}=-\,{k}_{3}{A}_{m}+{k}_{3}{A}_{i}$$

1d $$\frac{d{A}_{p}}{dt}=-\,{k}_{3}{A}_{p}+{k}_{3}{A}_{m}$$

1e $$\frac{d{G}_{m}}{dt}={k}_{4}{G}_{p}-{k}_{4}{G}_{m}$$

1f $$\frac{d{G}_{i}}{dt}={k}_{4}{G}_{m}-{k}_{5}{G}_{i}$$

2a $$\frac{d{G}_{p}}{dt}={k}_{1}{G}_{E}{G}_{p}-\,{k}_{4}{G}_{p}-{k}_{2}{A}_{p}{G}_{p}$$

2b $$\frac{d{A}_{i}}{dt}=-\,{k}_{3}{A}_{i}+\,{k}_{5}{G}_{i}$$

2c $$\frac{d{A}_{m}}{dt}=-\,{k}_{3}{A}_{m}+{k}_{3}{A}_{i}+{k}_{5}{G}_{m}$$

2d $$\frac{d{A}_{p}}{dt}=-\,{k}_{3}{A}_{p}+{k}_{3}{A}_{m}$$

2e $$\frac{d{G}_{m}}{dt}={k}_{4}{G}_{p}-{k}_{4}{G}_{m}-{k}_{5}{G}_{m}-{k}_{2}{A}_{m}{G}_{m}$$

2f $$\frac{d{G}_{i}}{dt}={k}_{4}{G}_{m}-{k}_{5}{G}_{i}$$

**Figure 2:**
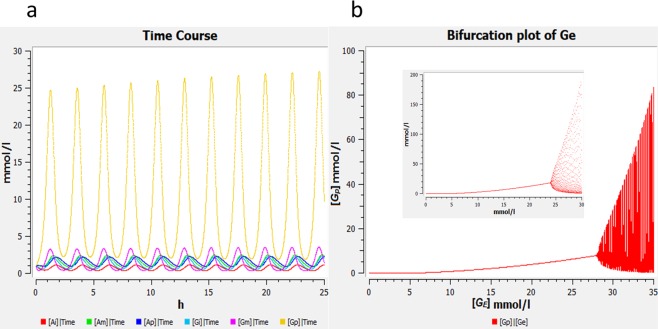
(**a**) Time course of ammonia (red, green and blue) and glutamate (cyan, magenta and yellow) in the interior, middle, and peripheral regions as computed by the c6ODE model. Oscillations are more spike-like than in the BM due to delay in the feedback induced by variable *G*_*m*_ and diffusion of ammonia. For parameters, refer to Table 1. **(b)** Bifurcation diagrams of *G*_*p*_ versus *G*_*E*_ for the c6ODE model and (inset) BM^11^. The supercritical Hopf bifurcation occurs at *G*_*E*_ = 28 mmol/l in the c6ODE (for BM bifurcation at *G*_*E*_ = 24.4 mmol/l) but qualitatively, the model has not changed. The two arms of the convex hull represent the amplitude of oscillation which widen as the value of *G*_*E*_ increases. The initial values of all variables were 1 mmol/l. Parameter values: refer to Table 1.

These two possibilities are modeled and analyzed separately. The results are shown in Fig. 2. For the bifurcation plot of the s6ODE model, see Fig. S1.

Simulating either of these model variants leads to results that do not show qualitative differences from the three-variable version^11^, except that the oscillations are more spike-like (Fig. 2a). This is because the middle layer causes a delay in the diffusion of glutamate and ammonia. A comparison of the bifurcation plots (Figs. 2b and S1) and the analogous Fig. 2b(inset) shows that there is no drastic effect on the Hopf bifurcation point nor the amplitude. Thus, we concluded that it is not necessary to go on adding further layers to the s6ODE or c6ODE variant until a smooth gradient is created, and it is adequate to use a three-variable system instead of an n-variable system. This is in support of our assumption that an ODE model instead of a PDE model is sufficient.

### Michaelis-Menten kinetics along with reversible reactions

In order to further improve the model by using biologically more realistic kinetics, we employ Michaelis-Menten kinetics to describe the uptake of glutamate from the environment, which is mediated by transport proteins embedded in the membrane of the cell^14^. Additionally, inspired by the reversible version^13^ of the BM^12^, we also make the following reactions reversible:

RMM $$\begin{array}{c}A+{G}_{p}\mathop{\mathop{\leftrightharpoons }\limits^{{k}_{-2}}}\limits_{{k}_{2}}A+biomass\\ {G}_{p}\mathop{\mathop{\leftrightharpoons }\limits^{{k}_{-4}}}\limits_{{k}_{4}}{G}_{i}\\ {G}_{i}\mathop{\mathop{\leftrightharpoons }\limits^{{k}_{-5}}}\limits_{{k}_{5}}A\end{array}$$

The consumption of *A* and *G*_*p*_ for protein (biomass) is considered reversible. This is biologically meaningful because biomass is subject to a permanent turnover, involving degradation to amino acids and ammonia. In the above reaction equation, we consider *A* on both sides for technical reasons. This formally implies that the stoichiometric coefficient of ammonia is zero, which is in line with the approximative assumption that the ammonia balance is almost exclusively affected by diffusion rather than by biomass turnover. Additionally, the diffusion of glutamate between the interior and periphery and the production of ammonia from glutamate is also reversible. The diffusion of ammonia to the surroundings is a rapid irreversible process^8^. Additionally, the uptake of glutamate from the environment is also irreversible since the process is mediated by transporters on the bacterial membrane^14^. Thus, the reversible Michaelis-Menten version of BM^11^, called model RMM is governed by the following equations:

RMM1 $$\frac{d{G}_{p}}{dt}=\frac{{V}_{max}{G}_{E}{G}_{p}}{{k}_{m}+{G}_{p}}-\,{k}_{4}{G}_{p}-{k}_{2}A{G}_{p}+{k}_{-2}A+{k}_{-4}{G}_{i}$$

RMM2 $$\frac{dA}{dt}=-\,{k}_{3}A\,+\,{k}_{5}{G}_{i}-{k}_{-5}A\,$$

RMM3 $$\frac{d{G}_{i}}{dt}={k}_{4}{G}_{p}-{k}_{5}{G}_{i}-{k}_{-4}{G}_{i}+{k}_{-5}A\,$$

**Figure 3:**
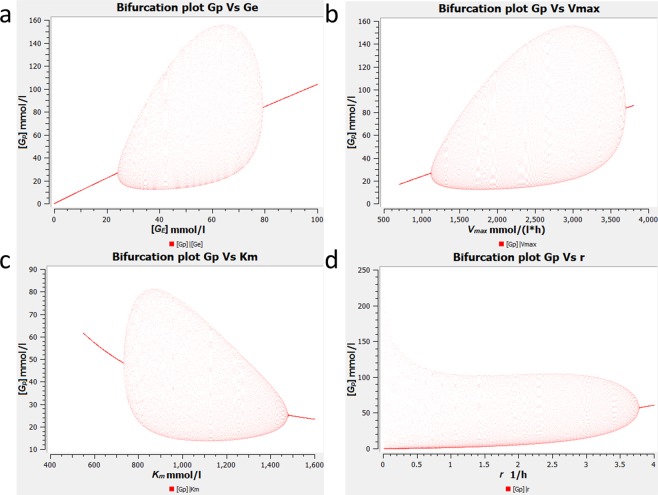
Bifurcation diagrams of *G*_*p*_ versus (**a)** *G*_*E*_ (**b)** *V*_*max*_ (**c)** *K*_*m*_ (**d)** *r*. In (**a–c**), we can observe a peculiar bubble-like pair of Hopf bifurcations. In the case of *K*_*m*_, the bubble is laterally inverted with respect to that of *G*_*E*_ and *V*_*max*_ indicating that increasing *K*_*m*_ implies a steep increase in amplitude at the left bifurcation whereas a gradual decrease at the right bifurcation. For parameter values refer to Table 1.

We then examine the sensitivity of the bifurcation to the parameters of the model. To simplify this, we assume *k*_*−*2_ = *k*_*−*4_ = *k*_*−*5_ = *r*. Note that for bifurcation analyses with respect to all parameters other than r, we use the plausible equality *k*_*−*2_ = *k*_*2*_, *k*_*−*4_ = *k*_*4*_ and *k*_*−*5_ = *k*_*5*_. Bifurcation plots for the parameters *G*_*E*_, *V*_*max*_, *K*_*m*,_ and *r* are shown in Fig. 3a–d. We used a wide scan range for each parameter because we do not have experimental values for all parameters. Once the parameters have been measured more accurately, their values can easily be included in the model.

For bifurcation parameter *G*_*E*_, we once again found the supercritical Hopf bifurcation just like in the BM. Interestingly, when *G*_*E*_ was further increased, we observed another bifurcation which was laterally flipped, supercritical and conjoined to the first bifurcation, so that the diagram resembles a ‘bubble’ (Fig. 3a). Such a bubble-like pair of Hopf bifurcations was also observed in several models of calcium oscillations^15^. The supercritical bifurcations indicate that there are certain values of *G*_*E*_ where the oscillations vanish with a smoothly decreasing amplitude. This implies that the oscillations will vanish with high availability of glutamate (nutrients), this was also found to be true experimentally^4^. The supercritical nature of the second bifurcation seems more biologically realistic than a switch-like transition (as would occur in a subcritical Hopf bifurcation) since the oscillatory process cannot suddenly halt. A similar effect is also observed when *V*_*max*_ is used as a bifurcation parameter instead of *G*_*E*_ (Fig. 3b).

A second Hopf bifurcation also occurs upon variation of the Michaelis-Menten constant, *K*_*m*_ (Fig. 3c). When *K*_*m*_ is very low, the saturation range is reached early, so that the kinetics becomes practically independent of *G*_*p*_. Therefore, no undamped oscillations can arise in that case. When *K*_*m*_ is very high, the slope of the kinetics is low, so that the feedback is not strong enough to enable undamped oscillations.

As can be seen in Fig. 3d, oscillations occur also if *r* is in the range of 2 h^−1^, similar to those of the forward reactions (see Table 1), which implies that oscillations can be observed when the rate constants of forward and reverse reactions are approximately equal. In our biological scenario, equal rates of forward and backward reactions are quite suitable.

### Michaelis-menten kinetics – fortifying the model to make it biologically plausible

Now we make two modifications to investigate the individual effects of either Michaelis-Menten kinetics or reversibility of reactions on this bubble-like bifurcation and determine which one of them are the cause of such a bifurcation.

We modify equation BM1 describing *G*_*p*_ as follows:

MMK1 $$\frac{d{G}_{p}}{dt}=\frac{{V}_{max}{G}_{E}{G}_{p}}{{k}_{m}+{G}_{p}}-{k}_{4}{G}_{p}-{k}_{2}A{G}_{p}$$

The other equations (MMK2 and MMK3) remain unchanged from the BM (eqs BM2 and BM3)^11^.

**Figure 4:**
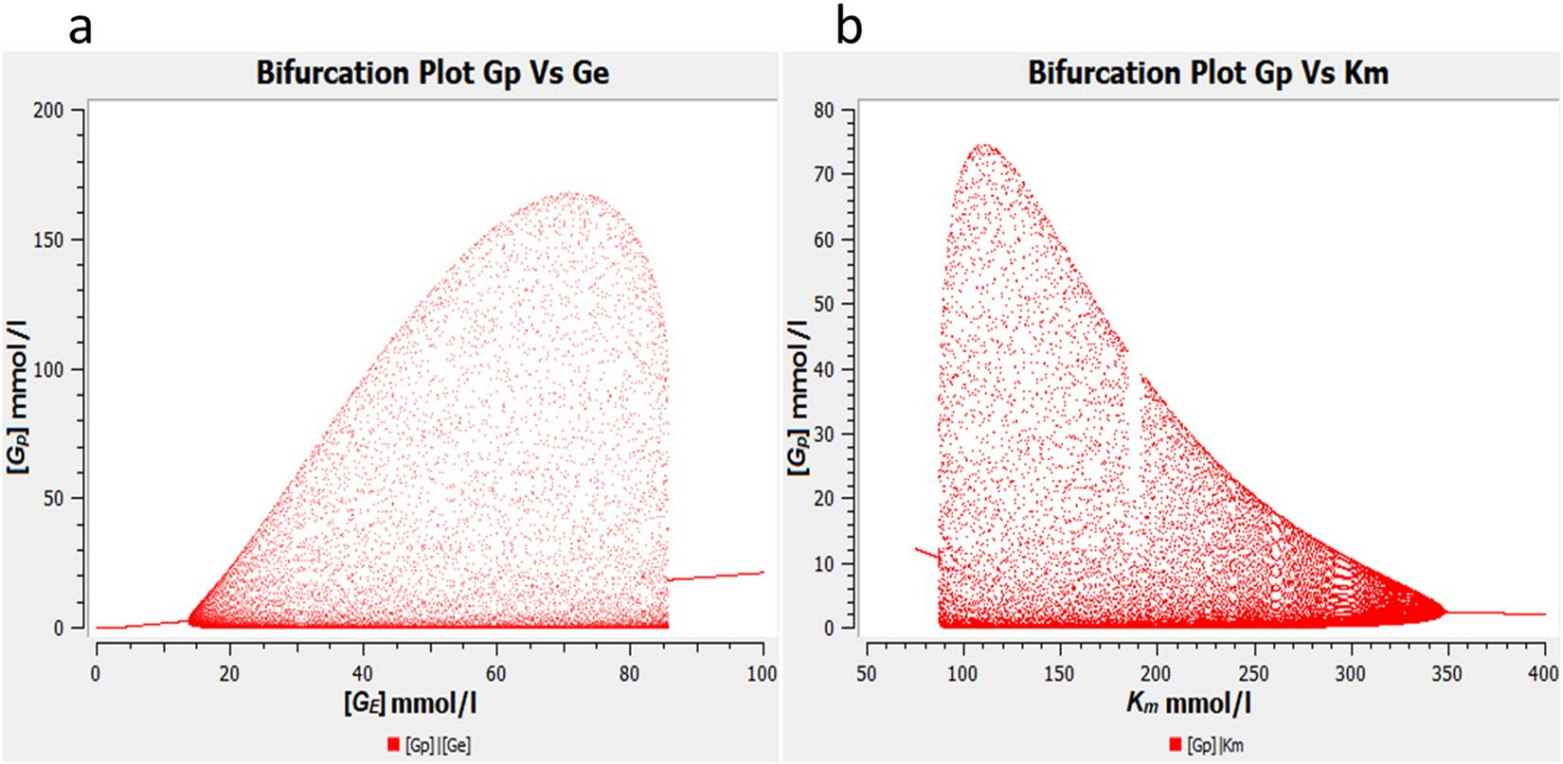
Bifurcation diagram of: **(a)** *G*_*p*_ versus *G*_*E*_. The same bifurcation bubble as for model RMM is obtained for *G*_*E*_ (and *K*_*m*_) with the only difference that the oscillations vanish in an abrupt way. Oscillations emerge gradually (supercritical bifurcation occurs at *G*_*E*_ = 13.6 mmol/l) and the bubble edges represent the amplitude of the oscillations which initially increases, then starts decreasing and eventually becomes zero (supercritical Hopf bifurcation at *G*_*E*_ = 85 mmol/l). Parameters: see Table 1, except: *K*_*m*_ = 150 mmol/l, *V*_*max*_ = 100 mMol-h^−1^. **b)** The bifurcation diagram is laterally flipped when *K*_*m*_ is used as a bifurcation parameter indicating an inverse relationship between amplitude and *K*_*m*_.

We found the same bubble-like pair of Hopf bifurcations that was seen for model RMM. This bifurcation could be explained as follows: If *G*_*E*_ gets very high, the glutamate input increases considerably, so that *G*_*p*_ increases up to the saturation range. Then, the kinetics becomes practically independent of *G*_*p*_, so that the essential properties of the minimal basic model are no longer granted and undamped oscillations can no longer occur (Fig. 4). While usually, the amplitude increases gradually at supercritical Hopf bifurcations, in our system, it increases quite abruptly in one of the bifurcations (Fig. 4a, right part of the bubble). A drawback of this model is the complete lack of reversible reactions which is unrealistic especially in the case of diffusion.

### The effect of reversible reactions

In the final modification, we investigate the effect of reversible reactions alone. The model is similar to model RMM except that it uses mass action kinetics instead of Michaelis-Menten. Thus the equation RMM1 from above becomes:

R1 $$\frac{d{G}_{p}}{dt}=({k}_{1}{G}_{E}-\,{k}_{4}){G}_{p}-{k}_{2}A{G}_{p}+{k}_{-2}A+{k}_{-4}{G}_{i}$$

And the equations RMM 2,3 are as above:

R2 $$\frac{dA}{dt}=-\,{k}_{3}A\,+\,{k}_{5}{G}_{i}-{k}_{-5}A$$

R3 $$\frac{d{G}_{i}}{dt}={k}_{4}{G}_{p}-{k}_{5}{G}_{i}-{k}_{-4}{G}_{i}+{k}_{-5}A\,$$

Similar to model RMM, *r* is the rate constant of reverse reactions for the bifurcation analysis with respect to these reactions and *k*_*−2*_ = *k*_*−4*_ = *k*_*−5*_ = *r*. In the bifurcation analysis for the other parameters, we assume *k*_*−2*_ = *k*_*2*_, *k*_*−4*_ = *k*_*4*_ and *k*_*−5*_ = *k*_*5*_.

This is the reversible version of BM^11^, called model R. Note that Eqs. R1 and R2 are identical to equations RMM2 and RMM3, respectively.

**Figure 5:**
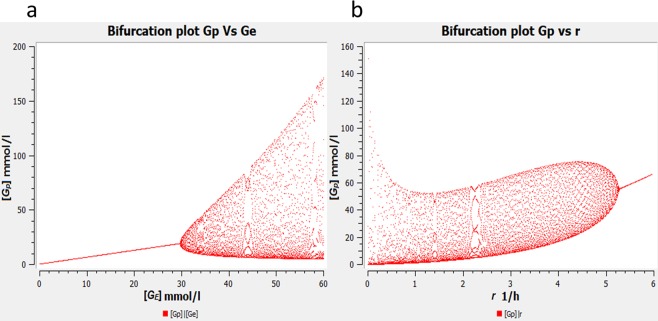
Bifurcation diagram of *G*_*p*_ (**a**) versus *G*_*E*_. *k*_1_ = 0.88 (mmol/l h)^−1^. Hopf bifurcation at *G*_*E*_ = 29.5 mmol/l (**b**) versus *r*: Oscillations are observed to diminish in amplitude overall as *r* increases but one can still see oscillations at *r* = 5.2 h^−1^. For further parameter values, see Table 1.

The system shows no change in the oscillatory behavior in comparison to the BM, in particular, bifurcation analysis with respect to *G*_*E*_ does not show the bubble-like Hopf bifurcation (see Fig. 5a). This indicated that Michaelis-Menten kinetics is the cause for the bubble-like Hopf bifurcation, in line with the explanation for that given above. However, when the reaction for the uptake of glutamate is made reversible, the bubble-like bifurcation can be seen once again (see Fig. S2). This is a new observation and was not anticipated by Wilhelm and Heinrich in their reversible model^13^ where they consider all reactions to be reversible with a rate constant of backward reaction 0.1 times that of the forward reaction.

The drawback of model R, however, is that it tends to a steady state when all the backward rate constants are equal to the forward rate constants (data not shown). This can be addressed and oscillations can still be obtained if the value of *k*_1_ is increased from 0.34 (mM* h)^−1^ to 0.88 (mM* h)^−1^ (Fig. 5b).

### Quasi-steady-state approximation for the model R

Since ammonia is a small molecule, it diffuses fast. Thus, the question arises whether a quasi-steady-state approximation^16^ can be applied. In the following, we analyze the special case where reaction 3 would be very fast. We could then assume that the variable *A* attains a quasi-steady state:

5 $$\frac{dA}{dt}=-\,{k}_{3}A+{k}_{5}{G}_{i}-{k}_{-5}A\cong 0,\,{\rm{which}}\,{\rm{leads}}\,{\rm{to}}\,A=\frac{{k}_{5}}{{k}_{3}+{k}_{-5}}{G}_{i}$$

From Eq. (5), we derive a reduced system:

6 $$\frac{d{G}_{p}}{dt}=({k}_{1}{G}_{E}-{k}_{4}){G}_{p}-\frac{{k}_{2}{k}_{5}}{{k}_{3}+{k}_{-5}}{G}_{i}{G}_{p}+\left(\frac{{k}_{-2}{k}_{5}}{{k}_{3}+{k}_{-5}}+{k}_{-4}\right){G}_{i}$$

7 $$\frac{d{G}_{i}}{dt}={k}_{4}{G}_{p}+\left(\frac{{k}_{-5}{k}_{5}}{{k}_{3}+{k}_{-5}}-{k}_{5}-{k}_{-4}\right){G}_{i}$$

The system shows two steady states:

8a,b $${G}_{{p}_{1}}={G}_{{i}_{1}}=0$$

which is the trivial steady-state (TSS)

9a $${G}_{{p}_{2}}=c\frac{({k}_{1}{G}_{E}-{k}_{4})({k}_{3}+{k}_{-5})}{{k}_{2}{k}_{5}\,}+(\frac{{k}_{-2}}{{k}_{2}}+\frac{({k}_{3}+{k}_{-5}){k}_{-4}}{{k}_{2}{k}_{5}})$$

9b $${G}_{{i}_{2}}=\frac{({k}_{1}{G}_{E}-{k}_{4})({k}_{3}+{k}_{-5})}{{k}_{2}{k}_{5}\,}+\frac{1}{c}\left(\frac{{k}_{-2}}{{k}_{2}}+\frac{({k}_{3}+{k}_{-5}){k}_{-4}}{{k}_{2}{k}_{5}}\right)$$

which is the non-trivial steady-state (NTSS)

where $c=\frac{{k}_{5}{k}_{3}+{k}_{-4}{k}_{3}+{k}_{-4}{k}_{-5}}{{k}_{4}({k}_{3}+{k}_{-5})}$ which is always positive.

The Jacobian matrix for the NTSS reads:

10 $${\bf{M}}=\left(\begin{array}{cc}({k}_{1}{G}_{E}-{k}_{4})-\frac{{k}_{2}{k}_{5}}{{k}_{3}+{k}_{-5}}{G}_{i}\, & \,-\frac{{k}_{2}{k}_{5}}{{k}_{3}+{k}_{-5}}{G}_{p}+\frac{{k}_{-2}{k}_{5}}{{k}_{3}+{k}_{-5}}+{k}_{-4}\\ {k}_{4} & \frac{{k}_{-5}{k}_{5}}{{k}_{3}+{k}_{-5}}-\,{k}_{5}-\,{k}_{-4}\end{array}\right)$$

For the TSS, it reads:

11 $${\bf{M}}=\left(\begin{array}{cc}{k}_{1}{G}_{E}-{k}_{4} & \,\frac{{k}_{-2}{k}_{5}}{{k}_{3}+{k}_{-5}}+{k}_{-4}\\ {k}_{4} & \,\frac{{k}_{-5}{k}_{5}}{{k}_{3}+{k}_{-5}}-{k}_{5}-\,{k}_{-4}\end{array}\right)$$

The eigenvalues are ${\lambda }_{1,2}\,=\frac{-b\pm \sqrt{{b}^{2}-4ac}}{2}$, where

12 $$\begin{array}{c}a=1\\ b=-\,{a}_{11}-{a}_{22}=-\,\left[{k}_{1}{G}_{E}-{k}_{4}+\frac{{k}_{-5}{k}_{5}}{{k}_{3}+{k}_{-5}}-{k}_{5}-{k}_{-4}\right]\\ c={a}_{11}{a}_{22}-{a}_{12}{a}_{21}=({k}_{1}{G}_{E}-{k}_{4})\left(\frac{{k}_{-5}{k}_{5}}{{k}_{3}+{k}_{-5}}-{k}_{5}-{k}_{-4}\right)-{k}_{4}\left(\frac{{k}_{-2}{k}_{5}}{{k}_{3}+{k}_{-5}}+{k}_{-4}\right)\end{array}$$

and for the NTSS (see supplement)

For the TSS: the eigenvalues are real if and only if ${b}^{2} > 4ac$ which can be achieved with *k*_1_*G*_*E*_ < *k*_4_. If the root value is smaller than the *b* value, then both eigenvalues are negative, so that the trivial steady state is a stable node. Otherwise, one eigenvalue is negative and the other one positive. The steady-state then is unstable, it is a saddle point. Furthermore, the case where ${b}^{2} < 4ac$ leads to complex eigenvalues and thus the steady-state is a stable focus (damped oscillations).

This implies that undamped oscillations cannot occur when ammonia diffuses so fast that it can be eliminated as a variable by the quasi-steady-state approximation. This also follows from general results by Hanusse^17,18^ saying a system involving two variables and only linear and bilinear terms cannot give rise to limit-cycle oscillations. As oscillations were observed in *B. subtilis* biofilms, we do not consider extremely high diffusion coefficients of ammonia in this study.

## Materials and methods

### Simulation

For computer simulations, we used the software COPASI versions 4.16 and 4.24^19^ and its LSODA deterministic solver. The simulations were double-checked using the Matlab ode15s (MathWorks) function. The figures of the simulations were produced using COPASI.

Except for *k*_2_, *k*_3,_ and *k*_4_, the rate constants were adopted from the publication of Wilhelm and Heinrich^12^ and were rescaled such that the period of oscillations matched the one in the experimental work by Liu *et al*.^4^. The rate constant of diffusion of ammonia, *k*_3_, is tuned to be twice as high as that of glutamate, *k*_4_. This is because the diffusion coefficient for ammonia^20^ is about 1.6E-05 cm^2^ s^−1^ while that for glutamate^21,22^ is about 8E-06 cm^2^s^−1^. The values of *K*_*m*_ and *V*_*max*_ are arbitrary because we just want to analyse the qualitative effect of enzyme kinetics, without a specific enzyme in mind.

## Conclusion

Here, we have analysed various model variants to describe biofilm oscillations. We used ODE systems, as is often done in mathematical biology^16,23^. Bocci *et. al*.^10^ used PDEs to describe biofilm oscillations, which is a computationally expensive approach. Our results above show that ODEs yield satisfactory results as well. ODE systems must involve at least two variables to describe limit-cycle oscillations^15,16,24^. When only linear and bilinear kinetics is used, at least three variables are needed^17,18^. Note that two-dimensional oscillator models such as the Brusselator^25^ and the Somogyi-Stucki model^26^ involve higher non-linearities. Therefore, our basic model is three-dimensional.

We have tested different modifications of the basic model^11^ and discussed salient features and disadvantages for each. All the models analysed here involve a positive feedback, notably of peripheral glutamate on its own uptake. In many biological systems, a positive feedback is the cause of oscillations^15,16,27^.

Adding additional layers makes the model more spatial. It may be possible to have more experimental inputs to the model. For example, the diffusion rates of ammonia and glutamate could be obtained from experiments to obtain more realistic amplitudes and wavelengths of oscillation. However, this does not qualitatively change the model. The bifurcation obtained is a supercritical Hopf bifurcation just like in the previous study^11^. We conclude that the modification of adding a middle region (models s6ODE and c6ODE) does not improve the model in any way. It highlights that ODEs describing just two layers are sufficient to model this biological phenomenon and that similar observations can also be obtained, if spatial effects are ignored.

The next modification, RMM, investigates the effect of Michaelis-Menten kinetics and reversibility. It has reversible reactions and the system shows undamped oscillations when the equilibrium constants of the reversible reactions are unity, which is realistic. It also uses Michaelis-Menten kinetics which is more robust to describe the process of nutrient uptake. Thus this modification is well suited to describe the periodic halting of growth of the *Bacillus subtilis* biofilm and has a greater scope to include experimental parameters like *K*_*m*_ and *V*_*max*_. We also obtain a peculiar bubble-like pair of Hopf bifurcations for this system. Such bifurcations were also obtained on models for cellular calcium oscillations^15^. The range beyond the second Hopf bifurcation is then called “overstimulation”, meaning that at high agonist levels, no oscillations occur^26,27^.

The RMM model predicts that if the nutrient supply gets high enough, oscillations will no longer continue (see Fig. 3a). In other words, metabolic oscillations are a mechanism to mitigate the nutrient limitation and therefore, can only be observed when nutrients are scarce. This observation, although quite intuitive, was not reported in the models of biofilm oscillations shown earlier^4,10,11,28^. This prediction could be validated in experiments, and knowing this threshold could help us control and modulate biofilm growth.

A similar bifurcation was also observed when *V*_*max*_ was used as a bifurcation parameter instead of *G*_*E*_. A second Hopf bifurcation also occurs upon variation of the Michaelis-Menten constant, *K*_*m*_ (Fig. 3c). When *K*_*m*_ is very low, the system reaches the saturation range early, so that the kinetics becomes practically independent of *G*_*p*_. Thus, the sole bilinear term is lost and the remainder linear system cannot show a limit cycle^16^. When *K*_*m*_ is very high, the slope of the kinetics is low, so that the feedback is not strong enough to enable undamped oscillations. The model also retains its minimality with respect to the number of variables and the number of bilinear terms used which makes it quite easy to analyze.

In order to investigate whether the cause of such bifurcation is the inclusion of reversibility or Michaelis-Menten kinetics, we analysed the two modifications separately in the further models. We also discussed the inspiration behind these individual modifications in the respective paragraphs.

The third extension, MMK, is achieved by using Michaelis-Menten kinetics for the uptake reaction. Uptake of glutamate has been shown to be mediated by a proton/glutamate symport protein in *B. subtilis*^14^. Here we neglect the diffusion of glutamate from the environment to the biofilm periphery but focus mainly on the uptake of glutamate by the cells in the periphery. We found that at one of the Hopf bifurcations, the amplitude increases very steeply. This could be considered physiologically advantageous because a pronounced division of labour is already reached close to the bifurcation. Analogously, in calcium oscillations, this phenomenon is beneficial for signal recognition^29^. For the system studied here, this modification led to a revelation that oscillations vanish after a high enough value of the bifurcation parameter. In terms of *G*_*E*_, it suggests that beyond a threshold value, the oscillations will no longer persist. This seems quite a likely case since oscillations arise in order to allow the interior of the biofilm obtain a steady input of glutamate. When glutamate is supplied in large excess, the interior will always have a steady supply of it and thus the oscillations will no longer be necessary. From a mathematical point of view, we observe that the saturation kinetics viz. Michaelis-Menten kinetics are crucial for obtaining such a bubble-like Hopf bifurcation. The major contribution of this model is that it identifies the cause of the bubble-like Hopf bifurcations.

The model R has already been inspired by a model established earlier^13^. We adopted and improved it by applying reversibility to only those reactions which are really reversible. For instance, the loss of ammonia is a highly irreversible reaction since ammonia is a small molecule that diffuses very rapidly once it is generated. From a physical point of view, all diffusion processes are reversible. However, when the concentration differences are large, the reverse process can be neglected. This was also done in models of calcium oscillations with respect to the calcium concentrations in the cytosol (low) and endoplasmic reticulum and external space (high)^26,27^. As the difference in glutamate concentrations between the peripheral and interior regions is less pronounced, we here extended the BM so that glutamate diffusion is reversible.

In model R, we found the same bubble-like bifurcation that was seen for the Michaelis-Menten model only when the reaction for the uptake of glutamate (given by the term *k*_1_*G*_*E*_*G*_*p*_) is made reversible (see Fig. S2). This was not anticipated by Wilhelm and coworkers^13^, who also proposed a fully reversible version of their model, and is thus an interesting new observation. Biologically it implies that when there are reversible reactions involved, there will be a stabilizing effect in the system that will prevent the amplitude to increase indefinitely with the bifurcation parameter. However, the uptake of glutamate is an active process and, therefore, can be modeled to be irreversible. In that case, only a single Hopf bifurcation is observed (Fig. 5a). The drawback is that the system tends to a steady-state (i.e. oscillations vanish) when all the backward rate constants are equal to the forward rate constants, which means that the equilibrium constants of the reversible reactions are unity. This can be resolved by increasing *k*_1_ indicating that a higher glutamate uptake rate is required for oscillations to persist at greater reversibility. Thus, this modification helps to correct the glutamate uptake rate *k*_1_. It also helps identify the reaction that causes the bubble-like Hopf bifurcation.

This work highlights the importance of simple ODE based models to describe the observations of Liu *et al*.^4^. Each modification presented here reveals a new piece of the puzzle which, when put together, will help us see the broader picture that is biofilm oscillations. The extended models presented here are still simpler and easier to handle than the Liu model (which involves 6 ODEs) and describe the experimental observations equally well. The aim of both the Liu model and ours is to describe biofilm oscillations qualitatively or at best semi-quantitatively. It is difficult to say whether the extended models describe the observations better than the basic model because no comprehensive parameter scan had been performed in experiment. As the extended models involve more parameters, it is likely that they can be fitted better to data obtained in the future.

The transition between oscillatory and stationary regimes can be gradual as in the case of model RMM or abrupt as in the case of model MMK which makes the BM flexible to describe two contrasting scenarios. An additional experimental investigation will provide more clarity about the exact nature of oscillations in the biofilm of *Bacillus subtilis*.

### Ethical approval and informed consent

Ethics approval was not required for this study and not applicable

## Supplementary information

Supplementary information.

## Data Availability

No data are associated with this article.
