# Supplementary material for: Extending the minimal model of metabolic oscillations in Bacillus subtilis biofilms
Source: Sci Rep. 2020 Mar 27;10:5579. doi: 10.1038/s41598-020-62526-6 (PMC7101430; doi:10.1038/s41598-020-62526-6)
Supplement: Supplementary file 1 — Supplementary information. [file 41598_2020_62526_MOESM1_ESM.docx]

**Supplement to:**

**Extending the minimal model of metabolic oscillations in
*Bacillus subtilis* biofilms**

Ravindra Garde ^1,2,4^ , Bashar Ibrahim ^1,3,4*^, Stefan Schuster ^1^*

^1^Department of Bioinformatics, Matthias Schleiden Institute, Friedrich Schiller University Jena, Ernst-Abbe-Platz 2, 07743, Jena, Germany. 2

^2^Max Planck Institute for Chemical Ecology Hans-Knöll Str. 8, 07745, Jena, Germany. 3

^3^Centre for Applied Mathematics and Bioinformatics, and Department of Mathematics and Natural Sciences Gulf University for Science and Technology, Hawally, 32093, Kuwait. 4

^4^These authors contributed equally: Ravindra Garde and Bashar Ibrahim.
*email: [bashar.ibrahim@uni-jena.de](mailto:bashar.ibrahim@uni-jena.de); [stefan.schu@uni-jena.de](mailto:stefan.schu@uni-jena.de)

***Quasi-steady-state approximation non trivial steady state***

The Jacobian matrix for the NTSS reads:

$\mathbf{M}=\left( \begin{matrix} \left( k_{1}G_{E}-k_{4} \right)-\frac{k_{2}k_{5}}{k_{3}+k_{-5}}G_{i} & -\frac{k_{2}k_{5}}{k_{3}+k_{-5}}G_{p}+\frac{{k_{-2}k}_{5}}{k_{3}+k_{-5}}+k_{-4} \\ k_{4} & \frac{{k_{-5}k}_{5}}{k_{3}+k_{-5}}- k_{5}- k_{-4} \end{matrix} \right)$

The eigenvalues are $\lambda_{1,2} =\frac{-b\pm\sqrt{b^{2}-4ac}}{2}$, where

for the NTSS:

$\begin{matrix} a=1 \\ b=-a_{11}-a_{22}= {-[k}_{1}G_{E}-k_{4}-{GG}_{i}+\frac{{k_{-5}k}_{5}}{k_{3}+k_{-5}}- k_{5}- k_{-4}] \\ c=a_{11}a_{22}-a_{12}a_{21}={(k}_{1}G_{E}-k_{4}-{GG}_{i})(\frac{{k_{-5}k}_{5}}{k_{3}+k_{-5}}- k_{5}- k_{-4})- k_{4}(\frac{{k_{-2}k}_{5}}{k_{3}+k_{-5}}+k_{-4}) \end{matrix}$

$\mathrm{and} {GG}_{i}=\frac{k_{2}k_{5}}{k_{3}+k_{-5}}G_{iSS}$ (always positive , and $k_{3}+k_{-5}\neq0$)


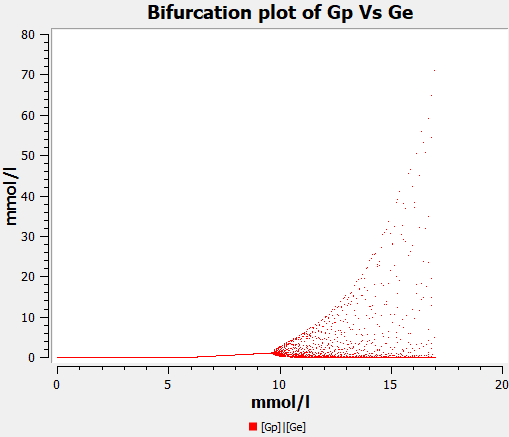


**Figure S1:** Bifurcation plot of *G_p_* versus *G_E_* (model s6ODE): The Hopf bifurcation occurs at about *G_E_* = 9.6 mmol/l, as opposed to 24.4 mmol/l for model BM or c6ODE.


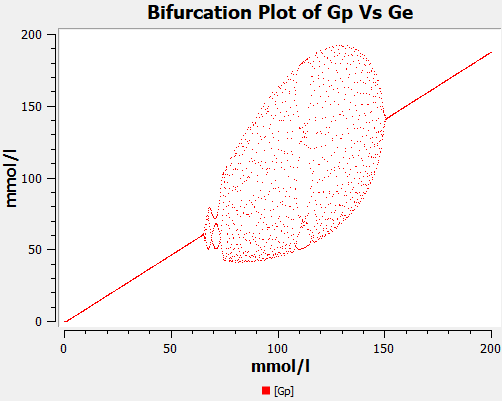


**Figure S2:** Bifurcation plot of *G_p_* versus *G_E_* (model R): Parameters: *k_1_* = 0.74 (mM* h)^-1^, *k_-1_* = 0.074 (mM* h)^-1^, *k_2_* = *k_-2_* = *k_5_* = *k_-5_* = *k_2_* = 2.3 h^-1^, *k_3_* = *k_-3_* = 4 h^-1^, *k_2_* = *k_2_* = 3 h^-1^. Only for this set of parameters, and when *k_-1_* is non zero, the bubble-like Hopf bifurcation can be observed, indicating that reversibility of reaction 1 is crucial for this kind of a bifurcation.
